# Changing the Office Design to Activity-Based Flexible Offices: A Longitudinal Study of How Managers’ Leadership Behaviours Are Perceived

**DOI:** 10.3390/ijerph192013557

**Published:** 2022-10-19

**Authors:** Johan Larsson, Stig Vinberg, Helena Jahncke

**Affiliations:** 1 Department of Occupational Health Sciences and Psychology, University of Gävle, 801 76 Gävle, Sweden; 2 Department of Human Work Science, Luleå University of Technology, 971 87 Luleå, Sweden; 3 Department of Occupational Health and Safety, Luossavaara-Kiirunavaara Aktiebolag (LKAB), 983 81 Malmberget, Sweden; 4 Department of Health Sciences, Mid-Sweden University, 831 25 Östersund, Sweden

**Keywords:** AFO, ABW, activity-based working, behaviour, flexible work, management, open-plan office

## Abstract

This longitudinal study examines the impact of office type on employees’ perception of managers’ leadership behaviours, which is an unexplored area. The expanding research related to activity-based flexible offices (AFOs) has mainly focused on employees’ working conditions and health outcomes, not on the changes in leadership behaviours when moving from traditional offices to AFOs. Office workers (*n* = 261) from five office sites within a large Swedish government agency were included in a controlled study of a natural intervention. At four sites, traditional offices were replaced by AFOs, while workers at one site with no relocation acted as the control. The same employees rated different leadership behaviours in a web-based questionnaire at baseline and at one follow-up. The analyses showed that relocations from cell and open-plan offices to AFOs were clearly related to a decrease in the perception of relation-oriented leadership behaviours. However, coming from open-plan offices to AFOs also decreased the perception of the other leadership dimensions. As expected, the control group was stable over time in their perceptions. This emphasises the need for organisations to provide managers with prerequisites so they can keep up with behaviours that support employees’ performance and health when office designs and ways of working are changed.

## 1. Introduction

In today’s working life there is a trend towards an office concept referred to as activity-based working [1] or activity-based flexible offices (AFOs) [2]. AFOs are flexible concepts which provide unassigned and shared work areas for different kinds of work. For example, rooms for concentrated work or meetings are often combined with open spaces with different work-zones and lounge areas [3]. Motives for this concept are that open spaces and flexible use of workstations may increase communication, which is expected to be beneficial for collaboration and performance among employees. Other motives are that working desks within AFOs can be more efficiently used by all employees, leading to less need of space and reduced costs, as well as the simple adaptation of work areas according to organisational changes [3]. Although there has been an increase in research regarding the effects of implementing AFOs during recent years, the results are somewhat contradictory. According to one systematic review [1], there is little evidence of AFOs affecting health-related outcomes. Some studies suggest that AFOs can enable employees to work more collaboratively as they engender efficient and effective communication, which may lead to increased productivity. However, studies also show that in some of the shared work areas, employees can have difficulty concentrating because of interruptions and high noise levels [1]. Hence, research on AFOs appears to have focused primarily on employees’ working conditions, satisfaction, and well-being (e.g., [1,4]), as well as productivity (e.g., [4,5,6]). To the best of our knowledge, there is little research considering the importance of perceptions of leadership behaviours related to AFOs, which is the focus of this study. 

There is an increasing number of studies indicating that leadership behaviours influence subordinate health. For example, associations have been found between leadership behaviours and job satisfaction [7], depression and lower back pain [8,9], stress and affective well-being [10], and self-rated health [11] among subordinates. However, to gain a deeper understanding of which behaviours are most strongly influencing subordinate health, and the situational variables that moderate and mediate in different populations, further research is needed. Relation-oriented leadership behaviours particularly (i.e., socialization and caring about subordinates) have been found to be related to employee health e.g., [11,12]. Research also shows an association between leadership behaviours and quality and efficiency outcomes in organisations. Associations between a combined style with relation and structure orientation and effectiveness have been reported in several studies e.g., [13,14,15]. In addition, studies show an association between leadership behaviours and quality outcomes e.g., [15,16,17]. 

When it comes to the implementation of AFOs, a systematic review [1] indicated that the majority of studies reported positive outcomes on productivity and work performance. However, other studies indicated decreased productivity, especially when moving from cell offices to AFOs [18,19]. One field study by Jahncke and Hallman [20] showed that objectively measured performance declined because of the increased noise levels (LAeq) in different work areas within the AFO. Performance declined most in the shared open-plan area where no noise restrictions were applied (i.e., an active zone). Therefore, it is important to define policies/rules for different zones and implement strategies for the new way of working [21]. It is here that the managers play an important role.

A review of research concerning activity-based working over the past ten years concludes that the short-comings of AFOs are related primarily to how this new way of working is implemented and how people use it, not to the concept itself. Three key components are important when implementing AFOs: physical environment, organisation aspects, and human perspectives. For a successful implementation of AFOs, a holistic approach is needed where subordinates are committed to the change and have flexibility to choose how and where to work, and to create a satisfactory physical and psychosocial work environment [22]. One study has also indicated that it is important that during the implementation of an AFO the manager is perceived to have change-oriented leadership behaviours to avoid productivity loss among employees [23]. This raises the question as to whether or not mangers will change and adapt new behaviours when the office concept is under transformation. However, there is a lack of research concerning how subordinates perceive their managers’ leadership behaviours within different office designs and whether these behaviours are perceived to change with a new office concept. Thus, the objective of this study, specified in the research questions, is to examine the effects of moving from traditional offices (cell offices, shared rooms, or small open-plan offices) to AFOs on employee’s perceptions of leadership behaviours in a large Swedish governmental organisation. The following research questions based on the PEO/PICO-model (population, exposure, intervention group, control group) are addressed:

1. In a large governmental organisation, do subordinate perceptions of their managers’ relation-, structure-, and change-oriented leadership behaviours differ before and after the change from cell offices or open-plan offices to activity-based flexible offices?
2. In a large governmental organisation, do subordinate perceptions of their managers’ relation-, structure-, and change-oriented leadership behaviours differ between those subordinates who experience the change from cell offices or open-space offices to activity-based offices and those who are in a control group (i.e., not changing office design) within the same organisation?

### Successful Leadership Behaviours

Leadership is a well-debated field in both theory and practice with a variety of definitions. The definition used in this study is ‘…an influence process used to accomplish organisational objectives’ (see [17] p. 5). In studies of leadership there are three main variables that are relevant to emphasise. These are the manager, the subordinate, and the situation where leadership takes place. In regard to the manager perspective, several different behaviour theories are used, such as the Full Range leadership model, including transformative, transactional, and laissez-faire leadership [24]; Leader-Member-eXchange (LMX) [25], and three-dimensional leadership behaviour theory [26,27,28]. During recent decades, Full Range leadership, and especially transformational leadership, have received substantial research interest in leadership studies. However, there are also critics of transformational leadership calling for a return to nascent and intermediary phases of theory development aimed at creating a stronger theory, better measures, and more actionable leadership models [29]. Another criticism is that the magnitude of interest and enthusiasm for transformational leadership is out of proportion given its weaknesses, e.g., conceptual limitations and the fact that the claim that transformational managers are more effective is not empirically supported [30]. The three-dimensional leadership behaviour theory, including the relation/employee, structure/task/production, and change dimensions, is decisively and empirically tested: two dimensions (relation and structure) in the 1940s and the third (change) introduced in the 1990s see [26,27,28,31,32]. The three-dimensional theory is used in this study because of its clarity, strong empirical support, and value when analysing different leadership styles and effects on the styles from different situation variables. A model that complements the three-dimensional behaviour theory is the healthy and effective leadership behaviour (HEL) model [16], which is based on studies of successful Swedish organisations. The model has been used in several studies e.g., [33,34] and includes nine groups of common leadership behaviours: a strategic and visionary leader role, communication and information, authority and responsibility, a learning culture, subordinate conversations, plainness and simplicity, humanity and trust, walking around, and reflective personal leadership [16]. When considering the three-dimensional leadership behaviour theory and the HEL model, high relationship orientation appears to be a universal component for successful leadership, while structure and change orientation vary in this regard depending on situational variables [16]. Furthermore, studies show that it is possible to influence the leadership behaviour of managers through various interventions e.g., [34,35,36].

The other main variable explored in this study, besides leadership behaviours, is the situation in which the manager practices leadership. Situation includes different aspects that can be used to describe an organisational setting, considering how different situational variables can moderate or mediate the effects of leadership behaviours on outcomes, and how different situations can influence perceptions of leadership behaviours. Following the manager’s authority in their leadership, the manager can change situational variables (e.g., structure of work, level of subordinate control over work, office designs) that influence outcomes (e.g., subordinate health), identifying the indirect mediating role that leadership behaviours have [37]. In this intervention study we focus on the situational variable of ‘office design’ and how changes in the design may possibly influence perceived leadership behaviours. The process of selection of office design change to AFOs, i.e., the indirect mediating effect of leadership behaviours performed by managers, is not included in this study.

## 2. Materials and Methods

This article uses empirical data from a project that started in 2015 considering office designs, specifically, an intervention regarding a change to AFOs. The project is a collaboration with a large Swedish governmental organisation: the Swedish Transport Administration. There are earlier studies published with data from this project that address other pre-stated research questions, and which provide a more detailed description see [20,23,38,39,40].

### 2.1. Design

This explorative quantitative study applied a case-control design with two intervention groups (i.e., employees working in cell offices or shared room/open-plan offices) and one control group. Four intervention offices and one control office were selected for the study. To measure possible changes in leadership behaviours, all subordinates completed a questionnaire three times between May 2015 and January 2017. The first was completed at the baseline point, the second three months after the intervention, and the third was a one-year follow-up study. In this article the results of the baseline study are only compared with the results from the study conducted one year after the intervention. This is because of measurement problems regarding the three-month study at one office site, which made the groups too small for running the intended analysis.

### 2.2. Data Collection

Data collection was performed at five office sites: the intervention groups (offices A–D) and the control group (office E). The included offices are located at different places in Sweden. Initially the senior management of the agency was contacted. Then a further dialogue regarding the study was held with the work environment specialists, office managers, and other key persons so that they could inform the employees of the up-coming study. A web-based questionnaire including measures of relation-, structure- and change-oriented leadership behaviours was sent to the employees by e-mail from the research group. The e-mail included information about the study as well as the link to the questions, and it followed all the ethical considerations, such as informed consent to participate. The inclusion criteria for participation were willingness to participate and being employed at any one of the five selected offices. Exclusion criteria were being on sick leave or maternal leave. Employees in the intervention groups that were not moving to AFOs, and/or employees that knew in advance that they were going to be changing jobs or retiring during the study, were also not included. Ethical approval of the study was given by the Regional Ethical Review Board in Uppsala Sweden (Dnr: 2015/118). All employees provided written consent before participating in the study.

The intervention group, including four offices (A–D), had two sub-groups. One group were sitting in cell offices (i.e., one person per office) and were moved to AFOs. The second group were in shared rooms (two or three persons per office) or small open-plan offices and were moved to AFOs. Because of the limited number of persons working in shared rooms, which would be too few for further analysis, the shared rooms and small open-plan offices were put together in one main category called open-plan offices. Arguments within the organisation for changing the work situation in the intervention groups were: to enable greater flexibility in where and how to work, a need for more efficient use of work desks (many desks were empty when meetings and work were located elsewhere), and to create opportunities for increased physical variation during the worktime.

The AFOs were organised so that each person in the office did not have a fixed personal work desk. The number of office desks were fewer than the total number of employees at the workplace. Every person selected their workplace based on vacancy and work tasks. Separate rooms were offered in all AFOs: quiet rooms/zones, web-meeting rooms, meeting rooms, conversation rooms, and conference rooms. Areas and single rooms for telephone calls were also provided in some AFOs’ lounges (for more details, see [39]). The intervention was planned and implemented by the Swedish Transport Administration without involvement of the research group. In the control group no intervention took place and the employees continued to work in cell or small open-plan offices during the study period. Just before the last follow-up measurement in 2016–2017 the employees were informed by the managers in the organisation that they would also change to AFOs in 2018.

During May to September 2015 all employees (*n* = 901) at the four intervention offices and the control office were approached with the web-based questionnaire prior to relocation. To get a broad picture of the studied organisations, the questionnaire in the project was designed to include questions from different validated instruments that covered various themes; leadership behaviours was one of them. The whole questionnaire was validated in the early stages of the project with the speak-out loud method [41]. The response rate among the groups completing the questionnaire at baseline ranged from 46% to 70%. A total of 38 employees were excluded because of parental leave, sick leave, and ending employment. From September 2016 to January 2017, at the 12-month follow-up, all employees (*n* = 802) were once again offered the opportunity to answer the questionnaire. The response rate ranged from 60% to 76%. A total of 94 employees were excluded because of parental leave, sick leave, or ending employment before completing the questionnaire. Of the remaining 708 employees, there were 251 non-responders, 180 who lacked data from one time-period of measurement (i.e., either from baseline or 12-month follow-up), and 16 had prioritized seats at the AFOs (i.e., not using the concept as intended) and were excluded from further analysis. It was possible to make the within-person comparisons between measurements for 261 employees and answer the research questions.

### 2.3. Participants

Table 1 shows that there was a higher proportion of men than women in all the sampled groups. The majority of participants were aged between 49–63 years, except for participants in the intervention group moving from open-plan offices to AFOs. The majority in all groups have a university education. 

### 2.4. Measurements

In both the baseline and follow-up measurements, group variables were included, dummy-coded as follows by group: intervention from cell offices to AFOs = 1, intervention from open-plan offices to AFOs = 1, control from cell-to-cell offices = 0, control from open-plan offices to open-plan offices = 0. The variable office type had two categories: cell offices and open-plan offices (including the categories shared room and open-plan offices). In the test of differences between intervention groups and the control group, the background variables were coded as gender (male = 1, female = 0), education (university education = 1, no university education = 0), and age dichotomised at the median (24–48 years = 1, 49–63 years = 0). The three dependent variables of leadership behaviour dimensions, relation-, structure-, and change-orientation, were measured with three indices containing five questions per index. The behaviour factors from the CPE-model (change, production, employee) have been used in different studies in several countries such as Sweden, Norway, Finland, and the USA [7,17,26,27,42,43,44,45]. The factors with high loadings were selected for the questionnaire. Furthermore, a question of socialisation behaviour with the subordinates was added to the relation index from the HEL-model [16]. The importance of socialisation (also known as Management by Walking around) has been proven to be an important behaviour in successful leadership [16,46]. Also, this behaviour is interesting to evaluate when changing to AFOs because of the large change in situational spatial presumptions for the leadership and especially the socialisation. The factor has been included in an earlier leadership study [34]. Comparable factors in the relation, structure, and change indexes have been used in the TRC (task, relation, change) model [15,28]. The relation-oriented dimension contained questions regarding consideration, caring about subordinates as persons, delegating responsibilities and authority, and socialising with subordinates to build relations and trust. The structure-oriented index included having clear goals, giving clear instructions, being exact about following rules and principles, allocating work to groups and individuals in a clear way, and following up on performed work. The change-oriented index contained questions regarding whether they have new work methods, communicate their vision, encourage development, discuss new ideas and propositions, and start development projects. These leadership behaviour indices were measured with a six-category scale ranging from do not agree at all (1) to totally agree (6). The items were totalled to get separate index scores for the baseline and follow-up measurements. ‘∆ Relation index’, ‘∆ Structure index’, and ‘∆ Change index’ scores were calculated by subtracting the initial index score (2015 Relation index) from the second index score (2016/2017 Relation index). A constant (31) was added to get the ∆-indexes transformed into a scale that did not include zero. The reliability of the indexes regarding relation-, structure-, and change-orientation were measured with Cronbachalpha for the different groups and the analysis showed high and stable Cronbach alpha-values between 0.88–0.94 with a limited range (see Table 2).

### 2.5. Statistical Analyses

There were in total 261 subordinates included in the analysis. A total of 124 were from the first intervention group—cell to AFOs; 68 were from the second intervention group—from open-plan to AFOs; and 69 were in the control group—having the same office setting in both measurements, either cell or open-plan office. A non-respondent analysis was performed using Pearson’s chi-squared test for differences. This was used to test the background variables of gender and age within the groups that responded, and those who did not respond. The chi-squared tests gave non-significant results (*p* > 0.05). Further, 19 interviews with non-respondents were performed. The main reasons for not responding were lack of time, not sitting in an activity-based office, and that the questionnaire was lengthy. The reasons for not working in the AFO were that they travelled a lot, they only worked from home, or that they had their own room at the office because of personal reasons. In these cases, they thought their responses were not representative for the study and did not respond.

Pearson’s chi-squared test for differences with a continuity correction were carried out to evaluate the differences regarding the background variables of age, sex, and education between the intervention and control groups in the baseline measurement (see Table 3). Both the uncorrected and corrected results were analysed. The test was performed to analyse whether there were any significant differences between the groups that were especially important to control for in the regression analysis. In the baseline measurement in 2015, there were no significant differences between the intervention groups and control group for all three variables of gender, age, and education. Since the intervention and control groups are similar, the background variables will not be included and controlled for in the final linear regression.

## 3. Results

As expected, the mean values for the control group were similar at baseline and at follow-up measurement, showing no significant changes over time (see Table 4). According to the mean values, the intervention group that moved from open-plan offices to AFOs (*n* = 68) had the largest differences in perceived leadership behaviours (see Table 4). At the follow-up, this group’s paired samples *t*-test showed a significant reduction of relation- (*p* = 0.002), structure- (*p* = 0.020), and change-orientation (*p* = 0.005) (see Table 5). The intervention group that moved from cell offices to AFOs (*n* = 124) also had the largest difference in the relation-oriented leadership behaviours (see Table 4), showing a significant reduction at the follow-up in the AFOs (*p* = 0.038; see Table 5). The mean values also indicate a slight decrease of structure-oriented behaviours in the AFOs (see Table 4). However, this change was not significant (see Table 5). The change-oriented behaviours remained unchanged for this intervention group (see Table 5).

Table 6 shows a significant difference between the subordinates’ perceptions of leadership behaviours in the control group compared to the two intervention groups. There is a significant difference between the control group and the intervention group going from open-plan offices to AFOs relating to the delta variables of the managers’ relation and change orientation. There were no significant differences between the control group and the intervention group going from cell offices to AFOs.

## 4. Discussion

This study examined employees’ experiences of leadership behaviours when the office design was changed from cell or open-plan offices to AFOs. Although research about the effects of AFOs concerning employees’ working conditions and well-being has increased, few studies have investigated employee perception of changes concerning leadership behaviours. Research supports the importance of involvement from the managers when implementing organisational and physical environment changes [48]. Studies have also highlighted the importance of leadership behaviours in relation to the implementation of different office types [2,49,50]. In addition, the COVID-19 pandemic has pushed many workers away from their offices and into their homes. It can be assumed that this hybrid way of working will prompt organisations to adjust their offices more into activity-based working (ABW) [22]. However, in the decision-making process about whether ABW should be introduced, the challenges raised by Marzban and colleagues such as unclear rules and policies for flexible working, lack of support for IT facilities, a weak management system, and difficulties concerning communication between subordinates and employers, also need to be considered. 

The main findings of this study are that the employees in the intervention group that went from open-plan offices to AFOs rated relation-, structure- and change-oriented leadership behaviours significantly lower one year after the intervention. The employees in the intervention group that moved from cell offices to AFOs reported lower values concerning relation- and structure-oriented leadership behaviours, although only the relation variable was at a significant level. When baseline measurements were compared to follow-up measurements, the ratings of behaviours in the control group were stable with no significant changes. When comparing the degree of experienced changes in the intervention groups versus the control group, the OLS regression results showed that the group that moved from open-plan offices to AFOs had a significant reduction in their perception of relation- and change-oriented leadership behaviours. For the intervention group that moved from cell offices to AFOs, there were no significant changes concerning the three leadership behaviours in relation to the control group. 

According to Wohlers and Hertel [2], successful managers must adapt their leadership behaviours to the functional features of different office types. In the case of AFOs it is important that the managers show trust in employees, rely on employees, and do not try to control the employees in the way they may do in traditional offices. If managers show limited trust and try to limit the autonomy of employees, the consequences can include decreased well-being, job satisfaction, and job performance [2]. Bodin Danielsson and colleagues [49] showed that the employee’s perception of managerial leadership differs between different office types. Their study showed poorer ratings of leadership among employees in shared-room offices and in flex-offices compared to cell offices. The best ratings were found for medium-sized open plan offices. Eismann and colleagues [50] studied AFOs and, according to factors related to leadership behaviours, they found new challenges for managers such as fewer contacts with employees and problems with evaluation of employee performance. However, AFOs might also support team building as managers and employees can share the same workspaces and work in greater proximity than in traditional offices [50]. 

The fact that this study showed lower ratings in the intervention groups when employees moved to AFOs compared to the control group (which stayed in cell offices) may have several explanations. Other studies in Swedish organisations show that moving to AFOs decreased perceived performance [5,23], satisfaction with the office design [5], as well as work satisfaction and well-being among employees [51]. These negative effects for the employees may contribute to and be related to the perceptions of employees of lower values of leadership behaviours. Another explanation may be that it is more difficult for managers to perform strong relation-, structure-, and change-oriented behaviours in AFOs compared to traditional offices. If this explanation is correct, it is problematic because involvement and support from managers concerning the implementation of organisational changes are important for how the employees experience the intervention [46].

Interestingly, the employees moving from open-plan offices to AFOs experienced a greater reduction in perceptions of all dimensions of leadership behaviours compared to the group moving from cell offices to AFOs, for whom only the relation-oriented behaviours were perceived to be reduced. This result may be due to differences in perceived leadership behaviours related to different office types [49]. Bodin Danielsson and colleagues [49] also showed a pattern of poorer ratings for leadership in shared-room offices and better ratings in medium-sized open-plan offices compared to cell offices. They discussed how a physically absent manager appears to be more problematic in shared-room offices than in other office types because independent subcultures could evolve. It is possible that these circumstances also influence the low ratings for leadership behaviours by employees when moving to AFOs. Perhaps it is also more difficult for both the manager and the employees to find each other and socialise when the work desks are unassigned and people work flexibly from different places. However, this needs to be explored in further research.

To summarise, the results of this study show lower employee ratings regarding leadership behaviours one year after the intervention when employees had moved from cell and open-plan offices to AFOs. In addition, this pattern is clearer among the latter group. The results are problematic because extensive research shows that relation-, structure-, and change-oriented leadership is important for well-being, working conditions, and organisational outcomes such as productivity and quality. In particular, a strongly pronounced relation-oriented leadership behaviour can be seen as a universal component impacting subordinate health and job satisfaction [16]. Structure- and change-oriented leadership behaviours can be seen as being of particular relevance when carrying out organisational and office design changes because of the large changes in the environment where the work is performed. The results point to the need for manger-focused interventions when changing traditional office designs to AFOs, including the knowledge gained from earlier leadership interventions e.g., [34,35,36,52].

### 4.1. Strengths and Limitations

Earlier research involving longitudinal studies about how employees experience changes of leadership behaviours when moving from cell or open-plan offices to AFOs is limited. A strength of this study is that it includes two intervention groups and one control group from the same organisation which are measured both at baseline and at one year follow-up. The inclusion of several office sites in different parts of Sweden increases the generalisability of the results and allows a comparison of the effects of implementing an AFO concept in different settings. It is, however, also important to note that one office category may contain many variations in the types of work environments that are actually provided at each company, and how well the office design fits the work tasks that are performed within that organisation and department.

Another strength is the use of validated questionnaires for measuring leadership behaviours of relation, structure, and change orientation. Although the inclusion of a control group and intervention groups from the same organisation make it possible to have some contextual factors under control, the results should be carefully generalised to other organisations. This is because the study is restricted to only one large Swedish organisation. In addition, longitudinal studies over longer periods might be needed if the changes of leadership behaviours are permanent or change over time. Although the study focuses only on managers and subordinates that answered the questionnaire at both measuring points, a limitation is that we have not measured the managers’ positions before and after the intervention on a detailed level. However, based on the information collected regarding the measured population in this study concerning leadership behaviours, the change in the number of included managers is low which can indicate stability. There were only two more managers after compared with before the intervention, 31 versus 33. 

### 4.2. Future Research

The findings of this study point to the need for both quantitative and qualitative research studying mechanisms beyond the changes in perceptions that employees have of leadership behaviours when implementing AFOs. More studies are needed of these issues in different types of AFOs and of the successful factors in implementation of AFOs and leadership interventions in this context. Particularly, more studies are needed to examine whether leadership training of managers before implementing AFOs is a way to avoid the negative effects on subordinates’ perceptions of leadership behaviours.

## 5. Conclusions and Implications

Changes from cell and open-plan offices to AFOs are related to lower ratings among employees regarding relation-oriented leadership behaviours. This result is more pronounced among employees moving from the open-plan offices, who also experienced a reduction in structure- and change-oriented leadership behaviours in the AFOs. In comparison with a control group the results are more pronounced among employees moving from open-plan offices to AFOs. They perceived relation- and change-oriented leadership behaviours significantly lower than the control group. These results emphasise the need for organisations to support managers before and during changes from traditional offices to AFOs, as this provides the prerequisites to maintain behaviours that can support the performance and health of employees.

In the context of post-pandemic conditions, it can be assumed that hybrid work using AFOs will increase. Therefore, training for the managers should focus on knowledge about what research has shown concerning negative and positive effects of AFOs and successful strategies for implementing these environments. Implementation of AFOs without leadership training of the managers is not recommended based on the study findings. 

## Figures and Tables

**Table 1:** Percentages of subordinates by gender, age, and education for the intervention and control groups, *n* = 261.

2015 and 2016/2017 ^1^ (*n* = 261)				
	Total ^1^ *n* = 261	I ^2^ *n* = 124	I ^3^ *n* = 68	C ^4^ *n* = 69
Gender				
Male	56.3%	57.3%	55.9%	55.1%
Female	43.7%	42.7%	44.1%	44.9%
Age ^5^				
49–63 years	51.7%	52.4%	48.5%	53.6%
24–48 years	48.3%	47.6%	51.5%	46.4%
Education				
No university education	37.2%	36.3%	36.8%	39.1%
University education	62.8%	63.7%	63.2%	60.9%

^1^ Population of subordinates responding both at baseline 2015 and follow-up 2016/2017. ^2^ I = Intervention group (from cell offices to AFOs). ^3^ I = Intervention group (from open-plan offices to AFOs). ^4^ C = Control group. ^5^ Min-max is 24–63 years.

**Table 2:** Cronbach alpha for the relation-, structure-, and change-oriented indices for 2015 and 2016/2017 for the intervention and control groups.

	2015 ^1^				2016/2017 ^2^			
	(*n* = 261)				(*n* = 261)			
	Total	I ^3^	I ^4^	C ^5^	Total	I ^3^	I ^4^	C ^5^
	*n* = 261	*n* = 124	*n* = 68	*n* = 69	*n* = 261	*n* = 124	*n* = 68	*n* = 69
Relation index	0.90	0.90	0.87	0.92	0.89	0.91	0.89	0.85
Structure index	0.88	0.85	0.91	0.92	0.87	0.88	0.88	0.86
Change index	0.93	0.93	0.89	0.94	0.93	0.93	0.89	0.94

^1^ Population responding in 2015 and 2016/2017—responses in 2015. ^2^ Population responding in 2015 and 2016/2017—responses in 2016/2017. ^3^ I = Intervention group (cell offices to AFOs). ^4^ I = Intervention group (open-plan offices to AFOs). ^5^ C = Control group. Alpha values ≥ 0.8 in italics (criteria used from Field) [47].

**Table 3:** Chi-square tests for the background variables of age, gender, and education in a comparison between intervention and control groups at the baseline measurement in 2015.

2015 and 2016/2017 ^1^				
(*n* = 261)				
	df	X ^2^	*p*	*p_cc_* ^3^
Gender ^2^ 2015				
Group ^2^ (cell office to AFO/control sitting in cell office), *n* = 173	1	0.251	0.616	0.741
Group ^2^ (open-plan office to AFO/control sitting in open-plan office), *n* = 88	1	0.107	0.744	0.944
Age ^2^ 2015				
Group ^2^ (cell office to AFO/control sitting in cell office), *n* = 173	1	1.673	0.196	0.261
Group ^2^ (open-plan office to AFO/control sitting in open-plan office), *n* = 88	1	2.150	0.143	0.226
Education ^2^ 2015				
Group ^2^ (cell office to AFO/control sitting in cell office), *n* = 173	1	0.039	0.844	0.983
Group ^2^ (open-plan office to AFO/control sitting in open-plan office), *n* = 88	1	1.130	0.288	0.422

^1^ Population of subordinates responding in both baseline 2015 and 2016/2017. ^2^ Two scale steps. ^3^ Continuity corrected *p*-value.

**Table 4:** Mean and standard deviation for the relation-, structure-, and change-oriented indices for subordinates in 2015 and 2016/2017, *n* = 261.

2015 and 2016/2017 ^1^				
(*n* = 261)				
	Total	I ^2^	I ^3^	C ^4^
	*n* = 261	*n* = 124	*n* = 68	*n* = 69
Relation index 2015				
Mean	4.83	4.94	4.86	4.59
Std.dev.	0.89	0.87	0.81	0.96
Relation index 2016/2017				
Mean	4.63	4.77	4.46	4.54
Std.dev.	0.98	1.00	1.09	0.79
Structure index 2015				
Mean	4.13	4.11	4.23	4.05
Std.dev.	0.98	0.96	0.96	1.03
Structure index 2016/2017				
Mean	3.98	4.04	3.91	3.96
Std.dev.	0.96	0.98	1.01	0.88
Change index 2015				
Mean	4.22	4.26	4.28	4.10
Std.dev.	1.12	1.20	0.95	1.14
Change index 2016/2017				
Mean	4.11	4.26	3.86	4.10
Std.dev.	1.17	1.23	1.31	0.82

^1^ Population of subordinates responding in 2015 and 2016/2017; ^2^ I = Intervention group (cell offices to AFOs); ^3^ I = Intervention group (open-plan offices to AFOs); ^4^ C = Control group.

**Table 5:** Paired samples t-tests for the baseline and follow-up regarding relation, structure, and change indexes for subordinates in 2015 and 2016/2017, *n* = 261.

All Subordinates 2015 and 2016/2017 ^1^									
(*n* = 261)									
	I ^2^ *n* = 124			I ^3^ *n* = 68			C ^4^ *n* = 69		
	*df*	*t*	*p*	*df*	*t*	*p*	*df*	*t*	*p*
Relation index									
Baseline/follow-up	123	2.092	0.038 *	67	3.303	0.002 **	68	0.414	0.680
Structure index									
Baseline/follow-up	123	1.122	0.264	67	2.379	0.020 *	68	0.855	0.396
Change index									
Baseline/follow-up	123	−0.021	0.984	67	2.907	0.005 **	68	0.000	1.000

^1^ Population of subordinates responding in 2015 and 2016/2017. ^2^ I = Intervention group (cell offices to AFOs). ^3^ I = Intervention group (open-plan offices to AFOs). ^4^ C = Control group. * Significant (*p* < 0.05), ** Significant (*p* < 0.01).

**Table 6:** OLS regressions with the transformed (added constant 31) ∆ Relation, ∆ Structure, and ∆ Change dimensions as dependent variables, *n* = 261.

All Subordinates 2015 and 2016/2017 ^1^			
(*n* = 261)			
	*b*	*p*	*95% CI*
∆ Relation index			
Group ^2^			
Intervention: Cell to activity-based (1) vs. control (0), *n* = 193	0.615	0.370	−1.966–0.736
Intervention: Shared room and open-plan to activity-based (1) vs. control (0), *n* = 137	−1.768	0.034 *^,3^	−3.399–−0.138
∆ Structure index			
Group ^2^			
Intervention: Cell to activity-based (1) vs. control (0), *n* = 193	0.077	0.901	−1.135–1.289
Intervention: Shared room and open-plan to activity-based (1) vs. control (0), *n* = 137	−1.139	0.189	−2.847–0.569
∆ Change index			
Group ^2^			
Intervention: Cell to activity-based (1) vs. control (0), *n* = 193	−0.008	0.991	−1.392–1.408
Intervention: Shared room and open-plan to activity-based (1) vs. control (0), *n* = 137	−2.118	0.031 *^,4^	−4.038–−0.197

^1^ Population of subordinates responding in 2015 and 2016/2017. ^2^ Reference category ‘control group’. ^3^ R^2^ = 0.033/Adj. R^2^ = 0.026; ^4^ R^2^ = 0.034/Adj. R^2^ = 0.027. * Significant (*p* < 0.05).

## Data Availability

Supporting data are available by reasonable request (J.L. email: johan.larsson@qfromz.com and johan.larsson@lkab.com).
